# YOLO11-based detection of manometry sensors in video-fluoroscopy imaging for computer-aided multimodal assessment of swallowing

**DOI:** 10.3389/fradi.2026.1767875

**Published:** 2026-04-14

**Authors:** Dionne S. Brandsma, Manuel Maria Loureiro da Rocha, Lisette van der Molen, Maarten J. A. van Alphen, Michiel W. M. van den Brekel, Françoise J. Siepel

**Affiliations:** 1 Robotics and Mechatronics Group, University of Twente, Enschede, Netherlands; 2 Head and Neck Surgey and Oncology, Netherlands Cancer Institute, Amsterdam, Netherlands

**Keywords:** deep learning, impedance manometry, object detection, video-fluoroscopy, YOLO11

## Abstract

**Purpose:**

Accurate assessment of swallowing function is essential in the diagnosis and monitoring of dysphagia following head and neck cancer (HNC). The simultaneous analysis of video-fluoroscopy swallow studies (VFSS) and high-resolution impedance manometry (HRIM) offers a more comprehensive evaluation, reducing subjectivity in VFSS and improving anatomical context of HRIM in HNC patients.

**Methods:**

The inherently low pharyngeal pressures in post-treatment HNC patients hinder the analysis of HRIM. As such, this study proposes a deep learning method for the automatic detection of HRIM sensors in VFSS using a YOLO11-based detector, aimed at enabling the automatic delineation of manometric regions. Detection performance was evaluated on 268 frames from 8 HNC patients using a leave-one-patient-out cross-validation approach. EigenCAM-based heatmaps were produced to analyze the model’s attention patterns.

**Results:**

The model achieved 95.8% Precision, 97.4% Recall, 96.6% F1-score with minimal variation between folds. Under different noise levels and bolus-simulated obstructions, performance remained robust. Our method outperformed previous template-matching methods for manometric sensor detection in VFSS. EigenCAM visualizations confirmed consistent attention to catheter regions.

**Conclusion:**

The proposed YOLO11-based detector provides accurate and robust localization of manometric sensors in VFSS sequences to facilitate computer-assisted HRIM-VFSS fusion for objective swallowing assessment.

## 1 Introduction

Head and neck cancer (HNC), referring to malignancies in the oral cavity, pharynx and larynx (1), ranks as the sixth most prevalent type of cancer worldwide, according to the 2022 Global Cancer Statistics (2). Up to 50% of patients treated for HNC experience oropharyngeal dysphagia (OD), an abnormality of the swallowing function (3, 4), due to the anatomical location of the malignancies and the treatment modality. OD often appears post-treatment, e.g., due to radiation-induced fibrosis or post-surgical abnormalities (3, 5). However, tissue damage of the upper aerodigestive tract, caused by the malignancies itself, may also contribute to this impairment (6). Severe complications associated with OD include malnutrition, dehydration, aspiration pneumonia and airway obstruction (3, 7). OD also imposes significant social and psychological burdens on patients that reduce their quality of life, where patients commonly present symptoms of anxiety, low self-esteem, depression and social isolation (8, 9). Accurate assessment of the swallowing function is therefore essential in the diagnosis and monitoring of OD progression, as well as supporting the creation of well-informed rehabilitation plans.

Currently, clinical evaluation of OD primarily relies on interventional imaging techniques, such as video fluoroscopic swallow studies (VFSS) (10). During VFSS, patients swallow boluses mixed with a contrast agent to visualize the swallowing activity in the recorded fluoroscopy sequences (11). While it provides detailed visualization of the swallowing function, its interpretation is inherently subjective and highly dependent on the level of experience of the clinician (12, 13).

In contrast, high-resolution impedance manometry (HRIM) offers a complementary, quantitative assessment of the swallowing activity by measuring pressure and impedance variations using a sensorized catheter (14). However, in current clinical practice, HRIM requires manual selection of the primary swallowing event and delineation of manometric regions (i.e., velopharynx, mesopharynx, hypopharynx and upper esophageal sphincter) (15). This process of manual delineation is especially demanding in HNC patients, who often show anatomical abnormalities of the upper aerodigestive tract, reduced pharyngeal pressures and irregular impedance patterns, compromising the overall accuracy and consistency of HRIM interpretation (13, 14).

The simultaneous analysis of HRIM and VFSS, combining the quantitative measurements of HRIM with the detailed anatomical visualization that VFSS provides, could overcome limitations of both modalities (16). However, despite this complementary value, clinicians must still interpret HRIM and VFSS separately, which results in increased workload (17, 18). Moreover, the current combined approach does not address key challenges of HRIM analysis in HNC such as the delineation manometric regions. Using fluoroscopic video from simultaneous HRIM-VFSS examinations to delineate manometric regions can substantially reduce the influence that low or absent pharyngeal pressures in HNC patients have on swallow assessment quality, and it could make the analysis process easier (19).

In this work, we propose a robust deep learning framework for HRIM sensor detection in VFSS. By accurately localizing individual HRIM sensors in fluoroscopy frames, we facilitate the automatic spatial registration of manometric regions within the upper aerodigestive tract. This represents a key step towards an automated multimodal framework that supports quantitative, computer-aided assessment of swallowing dynamics for a faster diagnosis and more accurate monitoring of OD in HNC.

## 2 Materials and methods

### 2.1 Small-object detection in fluoroscopy imaging

Over the last decade, multiple studies concentrated on the automatic detection of fine-scale structures, such as guidewire endpoints and catheter tips, within fluoroscopic imaging. Although these methods often involve knowledge-based approaches directly applied to segmentation masks (20, 21), others incorporate dedicated landmark detection deep-learning modules. For instance, Li et al. (22) proposed a segmentation attention hourglass network and, in a later work, a keypoint localization region-based CNN (R-CNN) (23). Furthermore, Yao et al. (24) formulated a Siamese network strategy, while Wang et al. (25) used a heatmap regression network and Ma et al. (26) implemented a deep learning-based Bayesian filtering technique for endpoint localization.

Previous learning-based segmentation and endpoint localization methods have been proven effective for delineating and tracking catheters and guidewires, and identifying their tips during interventional procedures (27). However, while the context of these approaches shares visual similarities to manometric catheters in VFSS, our objective is multi-instance localization of discrete sensor positions rather than contour extraction or landmark detection. This makes object detection a more appropriate framework for the task. Among single-stage object detectors, the YOLO (“You Only Look Once”) series (28) has emerged as one of the most widely adopted frameworks in medical imaging (29). By omitting the region proposal stage of traditional two-stage detectors such as R-CNN (30) and SPP-Net (31), YOLO performs detection in a single forward pass, achieving faster inference and making it thus particularly suitable for real-time clinical applications (32). In fluoroscopy imaging, variants such as YOLOv5 have showed robust localization of electromagnetic tracking (EMT) sensors (33) and cardiac catheter landmarks (34). Additionally, YOLOv3 has also been used to define regions of interest as a pre-processing step for multi-instance guidewire segmentation, where it demonstrated effective detection of guidewire structures in fluoroscopy images (22).

While the clinical importance of capturing HRIM data alongside VFSS to provide complementary physiological and anatomical insights has been highlighted in recent clinical guidelines (16), only few studies have attempted to translate this concept to multimodal framework. The implementation of deep learning-based methods currently remains unexplored, where previous efforts that investigated the simultaneous analysis of HRIM and VFSS have exclusively relied on knowledge-based image processing pipelines for swallow detection (35), catheter detection (19) and sensor localization. Jell et al. (17), for example, proposed a template-matching method to detect manometry sensors in oropharyngeal-esophageal VFSS recordings. However, while it was effective in high-contrast imaging, their method was sensitive to variations in image quality due to radiation dose. To address this, Geiger et al. (18) targeted low-dose VFSS of the esophagus and introduced a template-matching approach which involved catheter localization prior to individual sensor detection. Although their method showed improved robustness against low-dose lighting conditions, accurate sensor detection was limited in cases of partial catheter occlusions and noisy conditions.

### 2.2 Datasets and annotation

VFSS and HRIM recordings were simultaneously acquired from 8 HNC patients of varying dysphagic severity at the Netherlands Cancer Institute (Amsterdam, the Netherlands) under institutional ethical approval (IRBd21-210, IRBd23-322). The examinations were performed by an experienced clinical team of speech-language pathologists and radiology technicians. The 3.3 mm HRIM catheter (*Solar GI*™ *K103659-E-1180-D*, Laborie®, Portsmouth, NH, USA) contained 36 pressure and 16 impedance sensors, and captured data at 20 Hz. It was inserted transnasally until the upper esophageal sphincter and was positioned according to established clinical guidelines (15). Fluoroscopy images were obtained using the *CombiDiagnost R90*™ system (Philips®, Amsterdam, the Netherlands) at 30 frames per second (fps) while patients swallowed boluses of different volumes and consistencies mixed with a contrast agent, following the International Dysphagia Diet Standardisation Initiative (IDDSI) consensus (36). Each swallow study captured the complete oropharyngeal trajectory from a lateral view for a clear visualization of the anatomy and catheter.

For each patient, a single-swallow sequence was acquired from the continuous fluoroscopy recordings. The eight extracted sequences had an average duration of 8.30 ± 4.23 s (249 ± 127 frames). Frames were manually annotated every 0.25 s using Labelbox© (San Fransisco, CA, USA), with bounding boxes drawn around all visible manometry sensors. The selected annotation interval reduced inter-frame redundancy while capturing complete swallowing dynamics, resulting in 268 annotated frames with a mean per-patient average sensor length of 36.8 ± 7.4 pixels. The annotations served as a ground truth (GT) for model training and for performance evaluation during inference.

### 2.3 YOLO11 architecture

**Figure 1:**
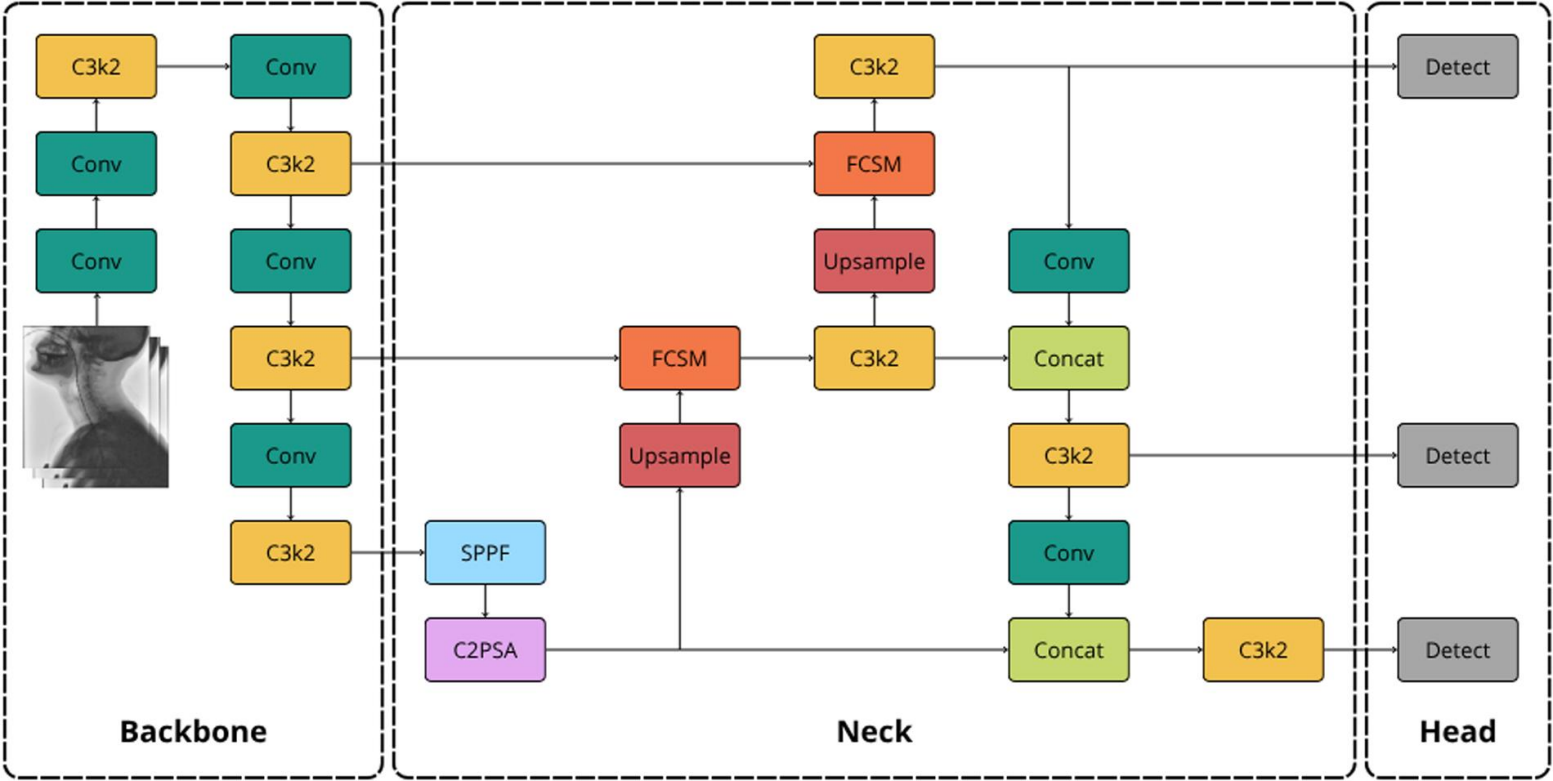
Schematic diagram of the YOLO11 architecture, highlighting its main components and flow.

YOLO11, the latest iteration of the Ultralytics’ YOLO series, was selected as objection detection framework for its high precision and improved processing speed compared to its earlier versions (37). At its core, the architecture of the YOLO11 remains equal to the three-stage design of its predecessors, comprising of a backbone, neck and head. YOLO11 improves the foundation laid by YOLOv8, replacing its C2f (faster implementation of the Cross-Stage Partial bottleneck with 2 convolutions) blocks throughout the network with the more computationally efficient C3k2 (Cross-Stage Partial bottleneck with 3 convolutions and reduced kernel size of k=2) block. Furthermore, while retaining the Spatial Pyramid Pooling—Fast (SPPF) block from previous versions for multi-scale feature aggregation, YOLO11 introduces the Cross-Stage Partial with Self-Attention (C2PSA) module right after the SPPF block for improved spatial attention in the feature maps (37). A schematic overview of the YOLO11 architecture is shown in Figure 1. Favouring high processing speed to enable real-time inference, the lightweight YOLO11-nano variant was chosen for this study.

### 2.4 Model training and cross-validation

The YOLO11n model was pretrained on the Common Objects in Context (COCO) dataset (38), covering 80 object categories. After initialization with the pretrained weights, the model was adapted to single-class object detection and was trained to individually detect all manometry sensors. Training was carried out over 100 epochs and early stopping was enabled with a patience of 25 epochs to prevent overfitting.

Due to the limited dataset size, model performance was evaluated using a leave-one-patient-out cross-validation strategy (39). Eight independent training rounds were performed, in which data from one patient were held out as the test set, while data from the remaining seven patients were used for training. In each training round, one patient from the training set was randomly selected as a validation patient. This allowed the monitoring of the training progress while enabling early stopping and ensuring that validation conditions closely resembled the final testing scenario.

The training hyperparameters were tuned on a randomly selected training fold using the Ultralytics tune tool for 100 iterations. As such, all images were normalized and resized to an input size of 640 × 640 pixels. Taking advantage of its generalization capabilities, the AdamW (Adam with Decoupled Weight Decay) (40) optimizer was used with a batch size of 16 and an initial learning rate of $1\times 10^{-3}$, which incrementally decreased using a cosine decay schedule to a factor of 0.01 of its initial value. Optimized hyperparameters are summarized in Table 1. For the remaining training hyperparameters, the default settings in the Ultralytics’ YOLO implementation were kept, including its standard loss function. Moreover, during validation and inference detections with a confidence score below 0.3 were filtered out and duplicate detections that overlap with an intersection-over-union (IoU) value over 0.5 were removed using non-maximum suppression (NMS). All training and evaluation was implemented using Python 3.10.12, PyTorch 2.3.1, CUDA 11.8, cuDNN 8.7, and Ultralytics 8.3.209 and was conducted on an NVIDIA A16 GPU with 15 GB of memory and 256 GB of RAM.

**Table 1:** Optimized hyperparameters.

Parameter	Value
Image input size	640 × 640 px
Batch size	16
Epochs	100
Patience	25
Optimizer	AdamW
Initial learning rate	0.001
Final learning rate factor	0.01
Learning rate scheduler	Cosine decay

### 2.5 Data augmentation

To improve model generalization and reduce biases towards specific fluoroscopy systems, several image augmentations were applied to VFSS frames in the training set. Due to the limited size of our dataset, data augmentations are essential to prevent overfitting of the model. These augmentations aimed to improve the diversity of the dataset and address possible variations in image appearance due to differences in radiation dose and imaging systems.

**Figure 2:**
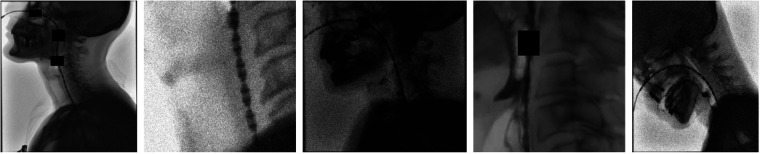
Examples of pixel-level augmentations, including dropout occlusions and contrast, brightness, blur and noise adjustments, applied to full-size frames and zoomed-in catheter regions to improve diversity of the dataset. Dropout augmentations consist of the black rectangles that mask regions of the catheter.

Image augmentations consisted of a combination of pre-training and online augmentations. Pre-training augmentations were applied to increase the size of the training and validation datasets in each fold by a factor of ten, prior to initializing the training loop. The pre-training augmentation pipeline began by randomly scaling each image by a factor between [1, 2] at a probability of 0.5. After scaling, a 450 × 450 crop was extracted from a region that contained the catheter, ensuring that the network always received a view of the relevant structure. These augmentations were followed by random brightness and contrast adjustments within the range [−0.3,0.3], Gaussian blur with $\sigma_{b}\in [0,2.0]$ and zero-mean Gaussian noise with standard deviation $\sigma_{n}\in [0.01,0.15]$, each applied with a probability of 0.5. To encourage spatial continuity of the sensor sequence, dropout augmentations were applied with a 0.75 probability of occurrence. These simulated bolus-induced catheter obstructions by randomly introducing up to three holes that masked-out catheter regions. All pre-training augmentations were implemented using Albumentations 2.0.8. Figure 2 shows five examples of these pixel-level, pre-training image augmentations.

Online augmentations were applied during training, in addition to pre-training augmentations, to further improve dataset variability. To promote spatial invariance, these augmentations comprised of geometric transformations, such as random rotations ($\left[\;-\;20^{\circ },20^{\circ }\right]$), translations ([−0.1,0.1]), and scaling ([0.7,1.3]), and were applied to all images during training. Additionally, images were flipped horizontally with a probability of 0.5.

### 2.6 Performance evaluation

Sensor detection performance and bounding box accuracy were evaluated using *Precision* (P), *Recall* (R), *F1-score* (F1), and Average Precision (AP) metrics across all patient folds. These metrics are defined based on true positive (TP), false positive (FP) and false negative (FN) detections (cf. Equation 1).

$$\text{P}=\frac{\text{TP}}{\text{TP}+\text{FP}},\;\text{R}=\frac{\text{TP}}{\text{TP}+\text{FN}},\;\text{F1}=\frac{2\times \text{P}\times \text{R}}{\text{P}+\text{R}}$$ (1)

A detection is considered true positive if there is sufficient overlap between predicted and GT bounding boxes. Overlap is quantified using the IoU metric (cf. Equation 2).

$$\text{IoU}=\frac{\left|\text{Intersection}\right|}{\left|\text{Union}\right|}$$ (2)

For the computation of *Precision*, *Recall* and *F1-score* an IoU threshold of 0.7 is used to determine true positive detections. In addition to P, R and F1, AP scores are computed, which are defined as the area under the *Precision*-*Recall* curve for a single object class (cf. Equation 3).

$$\text{AP}=\int_{0}^{1}\text{P}(\text{R})\;d\text{R}$$ (3)

We report AP@50, where the IoU threshold is 0.5, and AP@50-95, which averages AP over multiple IoU thresholds from 0.5 to 0.95 with increments of 0.05. Additionally, for all performance metrics, 95% confidence intervals (CI) were computed across patient-level scores using a t-distribution.

**Figure 3:**
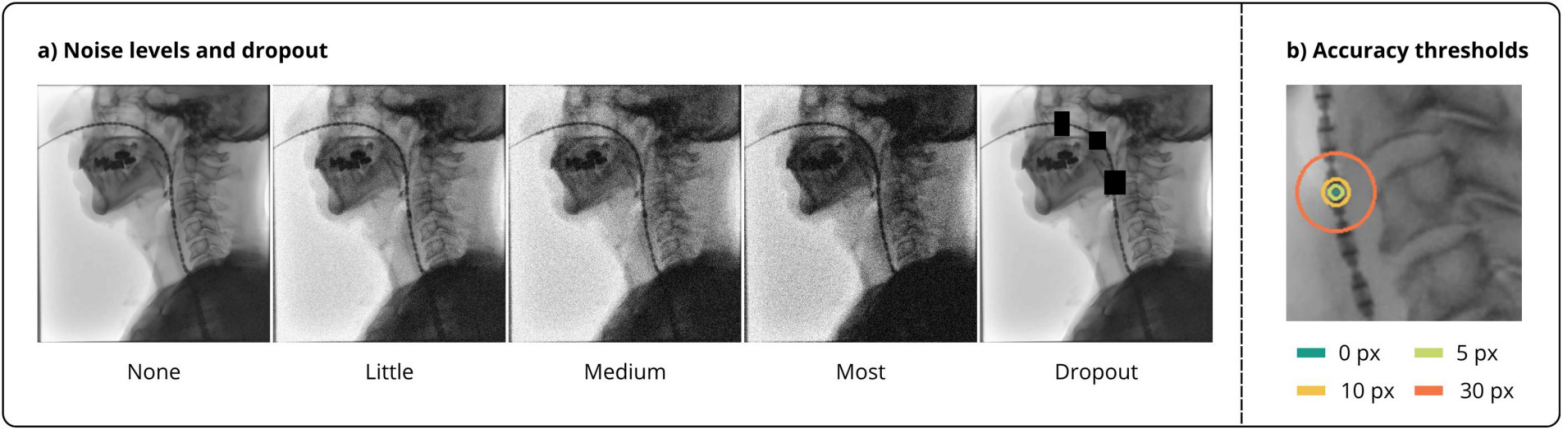
Visualization of the noise and accuracy levels used during evaluation. **(a)** Five noise conditions: *none*, *little*, *medium*, *most* and *dropout* **(b)** Distance-based accuracy thresholds. The circles with radii of 5, 10, and 30 pixels (1.35, 2.70, and 8.15 mm) represent the *exact*, *good*, and *acceptable* accuracy thresholds, respectively. A proportional correction factor was applied to the thresholds for different image scales.

For one-to-one comparison with Geiger et al. (18), we additionally computed *Precision*, *Recall* and *F1* using Euclidean distance thresholds between GT and predicted bounding box centers. Inference was conducted on unedited VFSS frames and three noise variants (“little,” “medium,” and “most”) with corresponding brightness, contrast and blurring adjustments to simulate different fluoroscopy radiation doses (cf. Figure 3). Table 2 shows the ranges of the transformations, conforming to the Albumentations implementation, that were randomly applied to images in the test set during evaluation. Although not included in the work of Geiger et al. (18), dropout augmentations were additionally introduced to assess robustness to partial catheter occlusions. Consistent with Geiger et al. (18), accuracy thresholds of 5, 10, and 30 pixels or 1.35, 2.70 and 8.15 mm (“exact,” “good,” and “acceptable”) were used. Patient-wise distance normalization was applied to the thresholds to account for the variation in average sensor distance within our dataset. Figure 3 illustrates the different noise levels, dropout occlusions, and distance-based evaluation thresholds.

**Table 2:** Transformation parameter ranges defining the *little*, *medium*, and *most* noise conditions.

Noise level	Brightness	Contrast	Gaussian blur	Gaussian noise
Little	[−0.1,0.0]	[0.0,0.2]	$\sigma_{b}\in [1.0,1.5]$	$\sigma_{n}\in [0.1,0.15]$
Medium	[−0.2,−0.1]	[0.2,0.3]	$\sigma_{b}\in [1.5,2.0]$	$\sigma_{n}\in [0.15,0.2]$
Most	[−0.3,−0.2]	[0.3,0.4]	$\sigma_{b}\in [2.0,2.5]$	$\sigma_{n}\in [0.2,0.25]$

### 2.7 EigenCAM with dynamic sign correction

In addition to quantitative measures, EigenCAM (41) was employed to provide qualitative visual explanations of the YOLO model’s predictions. Class activation maps (CAM) are a common tool for interpretation of CNNs by producing heatmaps that highlight regions of the image that are most important to the model’s decision, thereby visualizing the learned features of a model (42). Unlike gradient-based CAM methods, EigenCAM computes the first principal component of the activation maps using singular value decomposition (SVD) and does therefore not rely on backpropagation of gradients (41, 43). For YOLO11, EigenCAM was applied to convolutional layer 20, corresponding to the second C3k2 block directly attached to the detection head (cf. Figure 1). This block encodes medium-scale features that contribute to object detection. This layer was selected as the HRIM catheter (a thin, elongated structure) is better represented at this intermediate feature level rather than a coarser, deeper layer.

A known issue of EigenCAM is its tendency to erroneously emphasize background regions due to the sign ambiguity introduced by SVD calculations (43, 44). Therefore, we extend the standard EigenCAM approach with a dynamic sign correction mechanism as proposed by Liu et al. (43). Given the class activated output $O_{L=k}$, where the image is projected onto the last convolutional layer L=k, SVD is first computed by Equation 4, where *U* and *V* are orthogonal matrices and Σ is a diagonal matrix of singular values.

$$O_{L=k}=U\Sigma V^{⊤},$$ (4)

The preliminary EigenCAM localization map is then obtained with Equation 5, where *V*_1_ denotes the first principal component.

$$L_{EigenCAM}=O_{L=k}V_{1},$$ (5)

For consistent orientation of the localization map, dynamic sign correction ensures alignment of the primary feature information with the positive direction using Equation 6.

$$\begin{array}{rl} & L_{EigenCAM}^{*}=\left\{ \begin{array}{cc} L_{EigenCAM},\; & \text{if}\;|\max\left(L_{EigenCAM}\right)|>\\ \;|\min\left(L_{EigenCAM}\right)|,\;\\ -1\cdot L_{EigenCAM},\; & \text{otherwise.}\; \end{array}\right. \end{array}$$ (6)

By assuming a positive contribution of class-discriminative information, this correction mechanism effectively mitigates false emphasis on background regions and thereby improves the overall interpretability of the localization map.

## 3 Results

**Figure 4:**
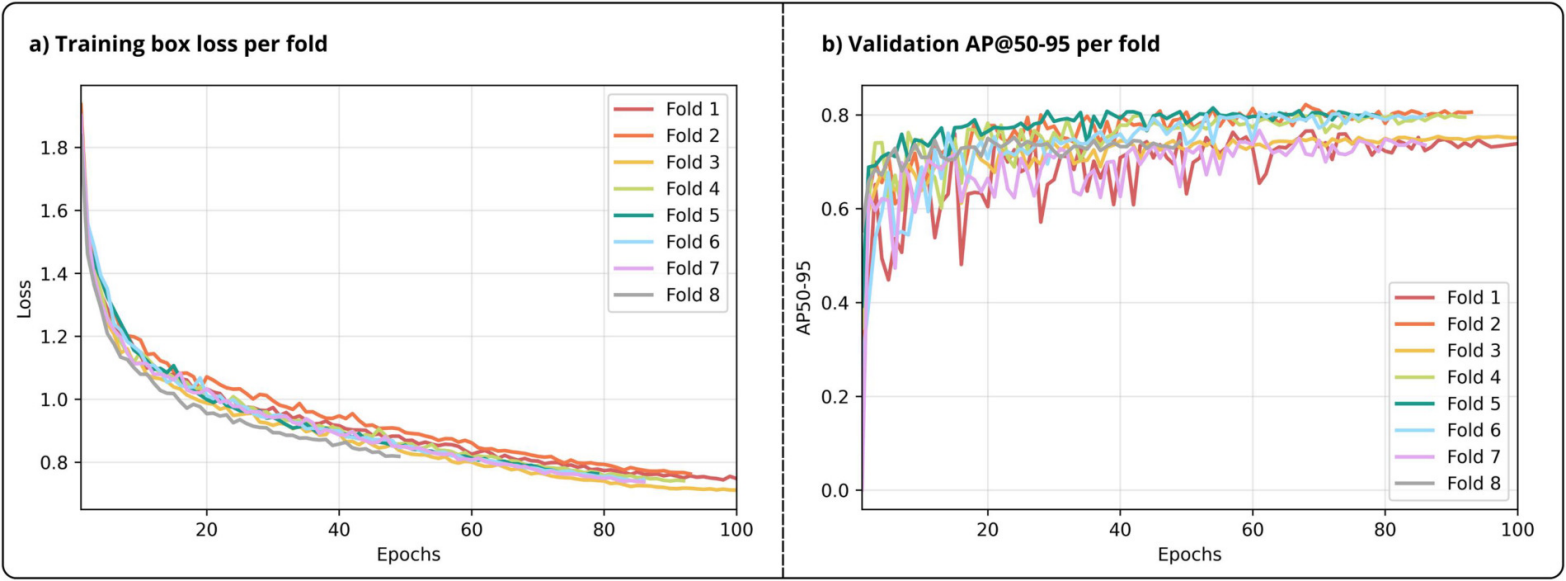
Training summary across all folds. **(a)** Training box loss across epochs per fold. **(b)** Validation AP@50-95 across epochs per fold.

Across all cross-validation folds, training progress of the YOLO11n model was evaluated using training loss and validation performance metrics. Figure 4a shows the training box loss, which is the primary component of the YOLO loss function in single class object detection quantifying bounding box accuracy. Additionally, Figure 4b shows the validation AP@50-95 score across epochs per cross-validation fold. The progression of the training box loss and validation AP@50-95 shows consistency in convergence between patient folds. The average number of training epochs was 86, where only two folds required the complete 100 epochs.

**Figure 5:**
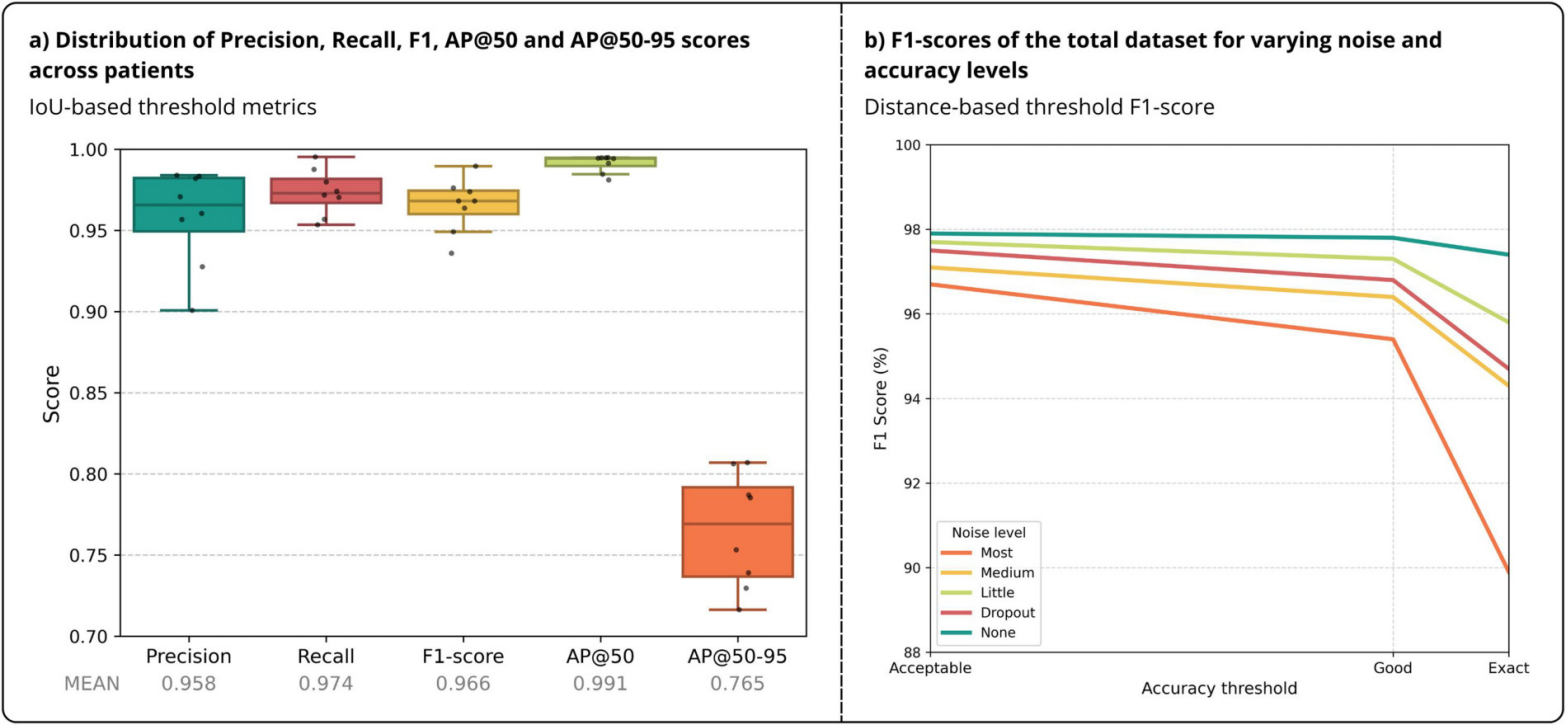
Sensor detection results across patients. **(a)** Boxplots of IoU-based threshold metrics (*Precision*, *Recall*, *F1-score*, AP@50 and AP@50-95) in unedited frames. Points represent performance instances of each patient fold. **(b)** *F1-scores* of the total dataset for varying noise conditions and distance-based accuracy thresholds.

During evaluation, the average processing time was 43 ms per image, which translates to 23 fps. As described in Subsection 2.6, evaluation metrics were computed using both IoU- and distance-based thresholds to assess bounding box and localization accuracy. As illustrated in Figure 5a, the model obtained a mean *Precision* of 95.8% (95% CI: 93.3–98.3%), *Recall* of 97.4% (95% CI: 96.1–98.6%) and *F1-score* of 96.6% (95% CI: 95.1–97.9%) at an IoU threshold of 0.7. The overall AP@50 and AP@50-95 reached 99.1% (95% CI: 98.6–99.6%) and 76.5% (95% CI: 73.6–79.5%) respectively on unedited frames. Moreover, as the inter-patient variation in these metrics was minimal, the model showed good model generalization across subjects.

Table 3 summarizes the average *Precision*, *Recall* and *F1-scores* and its standard deviation across patients with varying noise conditions and distance-based accuracy thresholds. Under no noise, the model achieved *F1-scores* of 98.2%, 98.1% and 97.7% for “acceptable,” “good” and “exact” accuracy thresholds respectively, demonstrating negligible sensitivity to increasing tolerance.

**Table 3:** Sensor detection results (mean ± standard deviation) across all patients measured in *Precision* (P), *Recall* (R) and *F1-scores* for different noise conditions and distance-based accuracy thresholds. Results from Geiger et al. (18) are shown in parentheses for comparison.

Noise	Accuracy	P (%)		R (%)		F1 (%)	
None	Exact	96.9 ± 2.7	(50.6 ± 20.8)	98.5 ± 1.3	(52.1 ± 19.6)	97.7 ± 1.2	(51.1 ± 19.9)
	Good	97.3 ± 2.6	(67.7 ± 19.4)	99.0 ± 1.2	(69.7 ± 16.8)	98.1 ± 1.2	(68.4 ± 17.7)
	Acceptable	97.4 ± 2.7	(85.2 ± 12.2)	99.0 ± 1.2	(88.3 ± 8.7)	98.2 ± 1.2	(86.4 ± 9.3)
Little	Exact	93.7 ± 6.8	(49.7 ± 22.5)	95.9 ± 4.4	(51.2 ± 20.8)	94.8 ± 5.5	(50.1 ± 21.4)
	Good	95.6 ± 4.2	(66.4 ± 21.6)	98.0 ± 1.9	(68.8 ± 18.1)	96.8 ± 2.8	(67.2 ± 19.5)
	Acceptable	96.2 ± 3.3	(84.4 ± 13.6)	98.6 ± 1.5	(88.4 ± 9.0)	97.4 ± 1.8	(85.8 ± 10.0)
Medium	Exact	91.4 ± 9.4	(47.7 ± 20.9)	93.7 ± 7.1	(44.9 ± 22.1)	92.5 ± 8.2	(47.7 ± 19.6)
	Good	94.5 ± 5.3	(65.4 ± 22.1)	97.0 ± 2.8	(61.9 ± 24.7)	95.7 ± 3.9	(65.6 ± 19.8)
	Acceptable	95.5 ± 3.8	(83.8 ± 14.9)	98.2 ± 1.8	(80.2 ± 24.3)	96.8 ± 2.4	(84.5 ± 11.3)
Most	Exact	86.3 ± 14.4	(38.5 ± 17.9)	88.2 ± 11.8	(29.7 ± 21.4)	87.2 ± 13.1	(37.7 ± 16.7)
	Good	92.9 ± 8.3	(56.7 ± 22.6)	95.2 ± 4.8	(43.8 ± 29.7)	94.0 ± 6.6	(55.6 ± 21.2)
	Acceptable	94.7 ± 5.7	(73.7 ± 26.6)	97.2 ± 2.1	(57.2 ± 37.8)	95.9 ± 3.8	(72.5 ± 25.2)
Dropout	Exact	94.1 ± 3.6	–	95.8 ± 2.0	–	94.9 ± 2.6	–
	Good	96.2 ± 2.9	–	97.9 ± 1.3	–	97.0 ± 1.7	–
	Acceptable	96.8 ± 2.5	–	98.6±1.1	–	97.7 ± 1.3	–

With a mild decline in metrics, performance remained robust when evaluated on increasingly noisy data and the model thereby demonstrated resilience towards variations in fluoroscopy quality. Even under the highest noise level (“most”) and strictest accuracy threshold (“exact”), the *F1-score* was 87.2%, a reduction of only 10.5% compared to unedited data. While mean *F1-scores* showed only small reductions under additional noise, the standard deviation increased notably at the highest noise condition. This suggests that noise sensitivity varied across patients.

The *F1-score* of the total dataset for varying noise levels at different distance thresholds is shown in Figure 5b. Across all noise levels, a steeper decline was observed between “good” and “exact” thresholds than between “acceptable” and “good,” indicating that while detections remained accurate, precise localization became more uncertain under noisier imaging conditions. During the dropout-based tests bolus occlusions were simulated. Here, the sensor detection performance also remained high, even in regions where the catheter was deliberately masked out. Although localization accuracy slightly reduced, with a more pronounced *F1-score* drop under the “exact” accuracy threshold, the results suggest effective interpolation of the sensor sequence when the catheter is partially obstructed.

**Figure 6:**
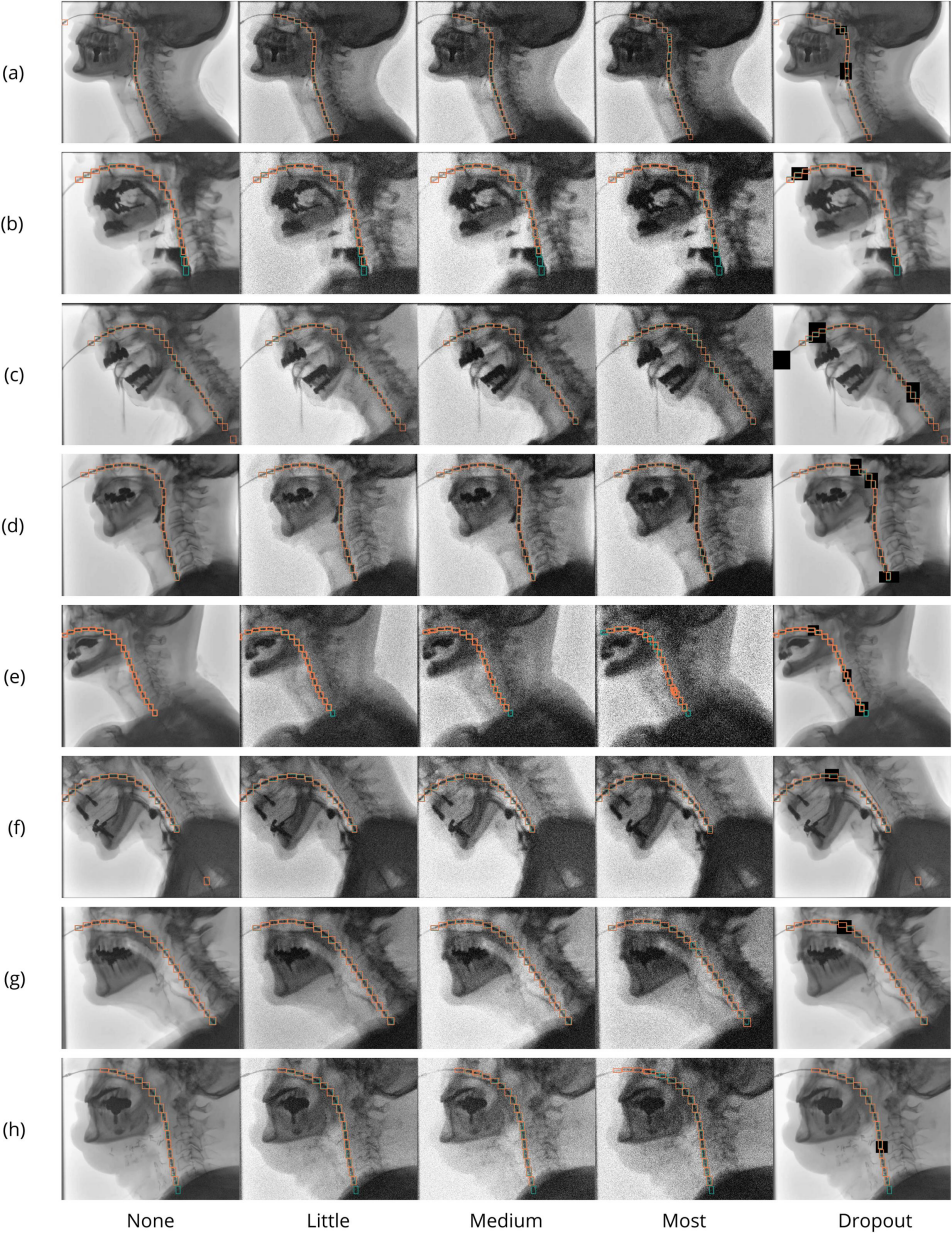
Several examples of sensor detection predictions. One representative frame of each subject **(a–h)** and noise condition (None-Dropout) is shown. The green bounding boxes represent the ground-truth annotation, while orange boxes are the model’s prediction.

Representative examples of sensor detection for each patient under varying noise conditions are shown in Figure 6. Qualitative evaluation showed that, occasionally, frames contain FP and FN detections, with increasing frequency in noisier conditions. Nonetheless, the majority of frames captured the full trajectory of the catheter and showed robust sensor localization.

**Figure 7:**
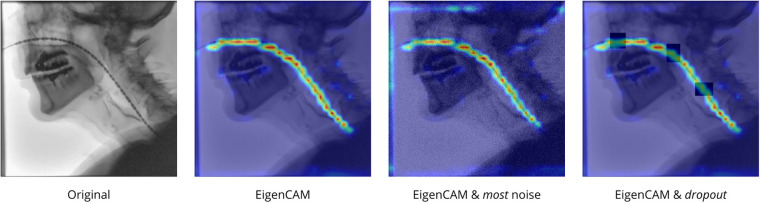
EigenCAM heatmap visualizations for the noise conditions *none*, *most* and *dropout*.

Additionally, Figure 7 presents EigenCAM visualizations for an example frame under “none,” “most” and “dropout” noise conditions. Regardless of noise condition, the activation maps consistently highlights the HRIM catheter with distinct local maxima near the center of individual sensor instances. Furthermore, spatial attention remains visually similar between “none” and “most” noise conditions, with only a minor decrease in peak intensities and mild edge artifacts under increasing noise. The EigenCAM heatmaps thereby acknowledge that the model’s feature extraction is largely invariant to image perturbations. In the “dropout” condition, the attention response is less strong within regions where the catheter is masked, but still maintains a continuous trajectory along the HRIM catheter.

## 4 Discussion

### 4.1 Performance evaluation and interpretation

In this study, we trained a YOLO11-based detector, which demonstrated accurate and consistent localization of manometric sensors in VFSS sequences across varying imaging conditions. Although the dataset comprised only eight patients, several methodological safeguards were implemented to mitigate overfitting and ensure reliable performance estimation. Model evaluation was conducted using leave-one-patient-out cross-validation, ensuring that each test set contained data from a completely unseen subject. The evaluation metrics exhibited narrow 95% CI across folds, indicating limited inter-patient variability and stable model performance. This consistency suggests that the model generalizes well within the current patient cohort despite anatomical and physiological differences between subjects. Finally, the low inference time (43 ms per image) supports the feasibility of real-time clinical deployment.

Although a direct comparison with the work of Geiger et al. (18) is not possible, as their study focused on lower-dose VFSS imaging of the esophageal region, we adopted their noise level and distance-based accuracy threshold convention to ensure the fairest possible comparison. In these conditions, the YOLO-detector outperformed the approach of Geiger et al. (18) under all conditions, as shown in Table 3. On noiseless frames, Geiger et al. (18) reported an average *F1-score* of 86.4% using a 30-pixel threshold (“acceptable”), which quickly declined to 51.1% when this accuracy threshold was restrained to 5 pixels (“exact”). For our dataset, the mean *F1-score* was 98.2% and 97.7% for “acceptable” and “exact” accuracy levels respectively. Similarly, in the strictest conditions (“most” noise and ’exact’ accuracy), we found a mean *F1-score* of 87.2%, while theirs was 37.7%. It is important to note that, compared to our dataset, the baseline frames of Geiger et al. (18) were of worse image quality due to its acquisition under low-dose fluoroscopy, resulting in a more challenging detection task to begin with. However, when comparing their most lenient conditions (“none” noise and “acceptable” accuracy) to our most demanding test condition (“most” noise and “exact” accuracy), our method still provides better results, achieving an *F1-score* of 87.2% compared to 86.4%. These results suggest that the YOLO-based detector outperforms the template-matching approach in all regards, showing reduced sensitivity to noise and improved localization accuracy emphasizing the advantage of deep learning—based detection methods over knowledge-based approaches.

A key advantage of YOLOv11 pipeline is spatial continuity of the sensor sequence, despite reduced localization accuracy, under both simulated (Figure 6, *Dropout* column) and real bolus occlusions (Figures 6b–d), demonstrating its ability to infer obstructed sensors. This is consistent with the EigenCAM heatmaps (Figure 7), where the complete trajectory of the catheter remains highlighted despite the weaker attention response in obstructed regions. The most common failure modes were FN or FP detections at endpoints (proximal or distal) of the catheter. These issues likely arise from mild overreliance on local contrast and edge cues at the beginning of the sensor sequence (Figure 6h) or from reduced sensor visibility at the distal end of the catheter (Figures 6a–c,e,h). Occasionally, FP arose from sensors that were not included in the GT annotations, such as reference (Figure 6a) or lower esophageal sensors (Figures 6c,f). These sensors are not clinically informative in the context of oropharyngeal HRIM–VFSS assessments and were therefore omitted from annotation. A substantially higher standard deviation in *F1-scores* under the most noisy condition (Table 3) was primarily due to one low-resolution video (Table 6e), exhibiting shorter sensor lengths (≈19 px, compared to 32–44 px), which disproportionately amplified the effect of added noise, reducing detection accuracy significantly.

### 4.2 Limitations and future directions

Despite the promising results, several limitations in this work remain. Since all recordings were acquired at a single institution using similar imaging protocols, the dataset size and diversity was limited. While beneficial, data augmentations can only reproduce real-world variability to a certain extend. To further assess the generalizability of this work, a multicenter study with expanded patient cohort would be required to include a broader demographic and varying fluoroscopy conditions.

In addition, this study is limited to frame-wise sensor detection and does not address temporal tracking or sensor-specific classification. For full integration with HRIM recordings, consistent sensor indexing across frames is required to reliably match VFSS-visible sensors with their corresponding pressure signals. Future work should therefore incorporate motion-aware temporal modeling into the current pipeline to preserve sensor identity despite partial catheter occlusions, patient motion, and prediction variability. Implementing these steps is essential for automated annotation of manometric regions and for improving HRIM analysis in HNC patients.

### 4.3 Final conclusions

This study presented a deep learning framework for automatic detection of manometric sensors in VFSS sequences using YOLO11. The model achieved high and consistent performance across patients with strong generalization and minimal inter-patient variation. Moreover, it showed real-time inference capability and maintained robust performance under variations in image quality and bolus-simulated occlusions. This supports its feasibility for clinical integration. Compared to previous template-matching methods, the YOLO11 detector provided higher accuracy and improved robustness, even in increasingly challenging conditions.

The results of this work demonstrate that our model has strong potential for deployment in real clinical workflows. By enabling real-time delineation of manometric regions directly within VFSS imaging, it addresses a key limitation of HRIM analysis in HNC patients, where anatomical alterations make manual region identification difficult and inconsistent. This approach could substantially streamline swallow assessment procedures and enhance the reliability of evaluations in clinically complex patient populations.

## Data Availability

The data analyzed in this study is subject to the following licenses/restrictions: The dataset is property of the NKI-AVL hospital. Requests to access these datasets should be directed to Manuel Maria Loureiro da Rocha, m.m.rocha@utwente.nl.
